# Identification of Submergence-Responsive MicroRNAs and Their Targets Reveals Complex MiRNA-Mediated Regulatory Networks in Lotus (*Nelumbo nucifera* Gaertn)

**DOI:** 10.3389/fpls.2017.00006

**Published:** 2017-01-18

**Authors:** Qijiang Jin, Yingchun Xu, Neil Mattson, Xin Li, Bei Wang, Xiao Zhang, Hongwei Jiang, Xiaojing Liu, Yanjie Wang, Dongrui Yao

**Affiliations:** ^1^College of Horticulture, Nanjing Agricultural UniversityNanjing, China; ^2^Horticulture Section, School of Integrative Plant Science, Cornell UniversityNew York, NY, USA; ^3^Institute of Agricultural Science of Taihu Lake DistrictSuzhou, China; ^4^Institute of Botany, Jiangsu Province and Chinese Academy of SciencesNanjing, China

**Keywords:** *Nelumbo nucifera*, small RNA, microRNAs, submergence, high-throughput sequencing

## Abstract

MicroRNAs (miRNAs) are endogenous non-coding RNAs with important regulatory functions in plant development and stress responses. However, their population abundance in lotus (*Nelumbo nucifera* Gaertn) has so far been poorly described, particularly in response to stresses. In this work, submergence-related miRNAs and their target genes were systematically identified, compared, and validated at the transcriptome-wide level using high-throughput sequencing data of small RNA, Mrna, and the degradome. A total of 128 known and 20 novel miRNAs were differentially expressed upon submergence. We identified 629 target transcripts for these submergence-responsive miRNAs. Based on the miRNA expression profiles and GO and KEGG annotation of miRNA target genes, we suggest possible molecular responses and physiological changes of lotus in response to submergence. Several metabolic, physiological and morphological adaptations-related miRNAs, i.e., NNU_far-miR159, NNU_gma-miR393h, and NNU_aly-miR319c-3p, were found to play important regulatory roles in lotus response to submergence. This work will contribute to a better understanding of miRNA-regulated adaption responses of lotus to submergence stress.

## Introduction

Lotus (*Nelumbo nucifera* Gaertn), a basal eudicot belonging to the plant family Nelumbonaceae, is a commercially important crop. In Asia, lotus is cultivated as an edible and ornamental plants and has high demand (Ming et al., 2013). It is also a source of herbal medicine because of strong antipyretic, antioxidant, anti-HIV, and anti-inflammatory properties. Due to its agricultural and medicinal importance, the genome of lotus has been sequenced and annotated recently (Ming et al., 2013; Wang et al., 2013), which offers an opportunity to identify and analyze genes in this species.

Global warming caused by human activities is making rain-induced flooding disasters more frequently and serious, which markedly affects plant distribution and crop yield. While lotus is an aquatic plant, it is sensitive to submergence due to rapid water level fluctuations. Submergence can inhibit photosynthesis, increase energy consumption, and ultimately lead to stunted growth and plant death (Fukao and Xiong, 2013). Thus, lotus can only grow in shallow regions near the shore of lakes and ponds as the abundance and distribution of lotus is largely influenced by water depth. An urgent need exists to improve the submergence tolerance of lotus varieties to address damage from flooding and to enhance the potential growing range within the littoral zone of lakes/ponds.

The regulatory mechanisms involved in plant submergence response have been characterized in some economically important semi-aquatic plant, i.e., deep-water rice (Nishiuchi et al., 2012). In deep-water rice, studies suggested the involvement of calcineurin B-like interacting binding kinase (CIPK) in coordinating submergence response, energy homeostasis, and growth in early shoot elongation under complete submergence (Bailey-Serres and Voesenek, 2010). The phytohormone, ethylene, which accumulates in submerged seedlings, plays a role in two divergent adaptive strategies, elongation (escape), and inhibition of elongation (quiescence). Several ethylene response factor (ERF) DNA binding proteins invoke the gibberellin (GA) pathway to promote shoot elongation in submerged seedlings (Bailey-Serres and Voesenek, 2010). The identification and functional characterization of two such *ERFs, SNORKEL* (*SK*) (Hattori et al., 2009), and *Submergence 1* (*SUB1*) (Xu et al., 2006), is a major advance toward breeding submergence-tolerant varieties. However, regulatory networks governing these genes and the overall response of lotus to submergence are poorly understood. In particular, the role of a group of recently intensively studied master regulators, microRNAs (miRNAs), in these regulatory networks were rarely studied (Lu et al., 2008; Bailey-Serres and Voesenek, 2010).

miRNAs are numerous small non-coding RNAs (20–25 nucleotides) that have recently emerged as important regulators in multiple biological processes, by guiding target mRNA cleavage or translational inhibition (Voinnet, 2009; Xu et al., 2013). In cells, small non-coding RNAs are produced via cleaving of their precursors which have a typical stem-loop structures (Bartel, 2004). DICER-LIKE 1 (DCL1) endonuclease recognizes and processes the stem-loop structure and forming miRNA:miRNA^*^ duplex. Then, mature miRNAs are released from the duplex by a helicase (Kurihara and Watanabe, 2004). miRNA^*^ strands which accumulate at low concentrations have been shown to be as important in regulatory networks as mature miRNAs (Basson, 2014). The mature miRNA or miRNA^*^ was incorporated by Argonaute (AGO) protein into an RNA-induced silencing complex (RISC) which guided cleaving at a specific position of protein-coding RNAs (Mallory et al., 2008; Pantaleo et al., 2010).

It is now well accepted that miRNAs play crucial roles in plant response to biotic and abiotic stresses (Sunkar et al., 2007). Altered miRNA expression levels in plants have been associated with plant tolerance of environmental stresses. Several stress-regulated miRNAs have been identified in model plant subjected to a variety of stresses, including: salinity (Sunkar et al., 2008), cold (Barakat et al., 2012), drought (Zhou et al., 2010), bacterial infection (Navarro et al., 2006), and heavy metal stress (Xu et al., 2013). Recent investigations have indicated that miRNAs are potentially involved in the regulation of adaptive response to hypoxia in rice (Paul and Chakraborty, 2013) and to submergence in maize (Zhai et al., 2013). High-throughput sequencing technology has significantly accelerated the discovery and functional characterization of miRNAs. Nevertheless, except for several model species, the expression patterns, and functions of most miRNAs are poorly understood in the other plant species (Zhao et al., 2012).

Although the population and abundance of miRNAs in lotus have been explored (Zheng et al., 2013; Hu et al., 2016; Pan et al., 2016; Shi et al., 2016), there are no reports on systematic identification and characterization of submergence-related miRNAs in lotus. In this study we determined the submergence response of miRNAs in lotus. We constructed and sequenced two small RNA libraries from lotus seedlings under control or submergence using Illumina sequencing technology. We identified and validated known and putative novel submergence-response miRNAs and corresponding target genes, and attempted to reveal complex miRNA-mediated regulatory network in lotus under submergence. These results provide insights into the molecular mechanisms underlying aquatic plant submergence responses.

## Methods

### Plant materials, growth conditions, and submergence treatment

Lotus (*Nelumbo nucifera* Gaertn) used in this study was grown under natural light conditions during May to August (Nanjing, China). For submergence treatments (Sub), 3-month-old lotus seedlings were transferred into a plastic tank and water was added into the tank to a water depth of 20 cm (above the top of plants). Plants without submergence treatment was considered as the control (Ck). After 12 h of submergence treatment, total RNA was isolated from whole seedlings using Trizol reagent (Invitrogen, USA) according to the manufacturer's instructions.

### Small RNA and mRNA-seq library preparation and illumina sequencing

For RNA-seq, three independent experiments with at least three replicates each were conducted and seedlings were sampled after treatments. Two sets of total RNA obtained from combined samples with (Sub) or without (Ck) submergence-treatment were used for cDNA library construction and sequencing (without replicates) at the Total Genomics Solution (TGS) company (Shenzhen, China), according to the manufacturer's instructions. Sequence data from this article have been deposited in the GenBank data libraries under accession numbers PRJNA354065 (Ck, SRR5035892; Sub, SRR5035891).

### Small RNA analysis

Overall workflow of TGS bioinformatics pipeline for small RNA libraries is shown in Figure S1. Raw reads (50 nt) obtained from Illumina sequencing were initially filtered using SOAPnuke to produce clean read. The data was processed via the following steps, removal of: (1) low quality reads and 3′ adaptor; (2) reads with 5′ primer contaminants and reads without the insert fragments; (3) reads without 3′ primers; (4) reads with poly A; (5) reads shorter than 18 nt. Next, clean reads were mapped to lotus genome (http://www.ncbi.nlm.nih.gov/genome/?term=nelmbo+nucifera) using the Bowtie2 program (v2.1.0) allowing no mismatch (Langmead and Salzberg, 2012). Perfectly matched reads were mapped to total plant miRNAs deposited in miRBase version 21 (http://www.mirbase.org) using Blast (v2.2.23) to identify known miRNA sequences. The following two criteria have to be met to define a known miRNA and its expression: (1) reads were aligned to the miRNA precursor in miRbase Version 21 (http://www.mirbase.org) with no mismatch; and (2) the produced reads were then aligned to the mature miRNA in miRBase with at least 16 nt overlap allowing offsets. Perfectly matched reads were also aligned to rRNA, snRNA, snoRNA, and tRNA deposited in GenBank (http://www.ncbi.nlm.nih.gov/genbank) and Rfam version 12.0 (http://rfam.xfam.org/), repeat sequences identified by RepeatMasker, and exons and introns annotated in the lotus genome using Blast (v2.2.23), with an *E*-value threshold of 1e-5. As some small RNA reads could be mapped to more than one category in the alignment, we followed the following priority rule: rRNA, snRNA, snoRNA, and tRNA (in which Genbank > Rfam) > known miRNA > repeat > exon > intron, to ensure every unique small RNA mapped to only one annotation. Small RNA reads which map to antisense exon, intron, or intergenic region of genome and the unannotated reads that did not map to any databases were used for novel miRNAs prediction using Mireap software (v0.2), with default parameters (Meyers et al., 2008). The expression abundance of miRNAs was normalized as transcripts per million (TPM) according to the following formula: Normalized expression = Actual miRNA count/Total count of clean reads) × 10^6^ (Chi et al., 2011). Following the normalization, the fold-change of miRNAs between Sub and Ck were calculated according to the formula: Fold change = log_2_(Sub/Ck) as described by Audic and Claverie (1997). The statistical significance of miRNA expression changes were estimated using the DEGseq R package v1.18.0 (Audic and Claverie, 1997; Wang et al., 2010). *P* value was adjusted using *Q* value (Storey and Tibshirani, 2003). *Q* <0.001 and log_2_fold changes ≥1 was set as the threshold for significantly differential expression.

### Prediction of miRNA targets and confirmation by degradome and transcriptome

Lotus mRNA sequences derived from lotus genome annotation (http://www.ncbi.nlm.nih.gov/genome/?term=nelmbo+nucifera) data was scanned for the presence of target sites of all identified miRNA sequences on the psRNATarget web server using default setting (http://plantgrn.noble.org/psRNATarget/) (Yin et al., 2008; Unver and Budak, 2009; Kantar et al., 2010; Dai and Zhao, 2011). Gene Ontology annotation for the target genes was performed using Blast2GO software suite (Conesa et al., 2005). Further, to uncover the biological function of these putative miRNA target sequences in cellular metabolic pathways, they were also annotated using KEGG automatic annotation server (Kanehisa and Goto, 2000).

To confirm targets of the miRNAs experimentally, a degradome (SRX1598165) of germinating lotus seedlings was analyzed (Hu et al., 2016). The raw reads were first cleaned by removing low-quality nucleotide reads and adapters using NGS QC Toolkit (v2.3.3) (Patel and Jain, 2012). Then the clean reads of the degradome, miRNA sequences, and predicted transcripts were used to confirm miRNA:target pairs using the CleaveLand pipeline (v4.3) with default parameters (Addo-Quaye et al., 2009).

The expression of corresponding target genes of the miRNAs was analyzed in our existing transcriptome data from lotus seedlings in response to submergence. The overall data analysis for mRNA libraries were summarized in Figure S2. Raw reads of both Ck and Sub libraries were first filtered with NGS QC Toolkit (v2.3.3) (Patel and Jain, 2012) to get clean reads. An index of the lotus genome sequence (deposited in NCBI) was built using Bowtie2 (v2.1.0) (Langmead and Salzberg, 2012) and clean reads were aligned to the reference genome using TopHat (v2.1.1) (Kim et al., 2013). Differential expression of target genes in response to submergence were calculated using the Cufflinks (v2.2.1) with default settings (Poelstra et al., 2015).

### RT-qPCR validation

For determination of miRNA expression, RNAs were reverse-transcribed by One Step PrimeScript miRNA cDNA Synthesis Kit (TaKaRa) and the cDNA was used for RT-qPCR analysis. U6 (Small nuclear RNA, snRNA) was used as an internal control. The forward primer for RT-qPCR validation of miRNAs was designed to match the candidate miRNAs, and the reverse primers were as provided by the kit. For determination of target gene expression, total RNAs were reverse-transcribed using an oligo(dT) primer and SuperScript Reverse Transcriptase (Invitrogen, USA). *Elongation factors 1* (*EF-1*) was used as an internal control for target gene expression analysis. RT-qPCR reactions were performed using a Mastercycler ep *realplex* real-time PCR system (Eppendorf, Hamburg, Germany) with SYBR Premix Ex Taq (TaKaRa) according to the manufacturer's instructions. The relative expression level was presented as values relative to corresponding control samples after normalization. The specific primers were listed in Table S1.

## Results and discussion

### High-throughput sequencing of small RNAs

**Figure 1:**
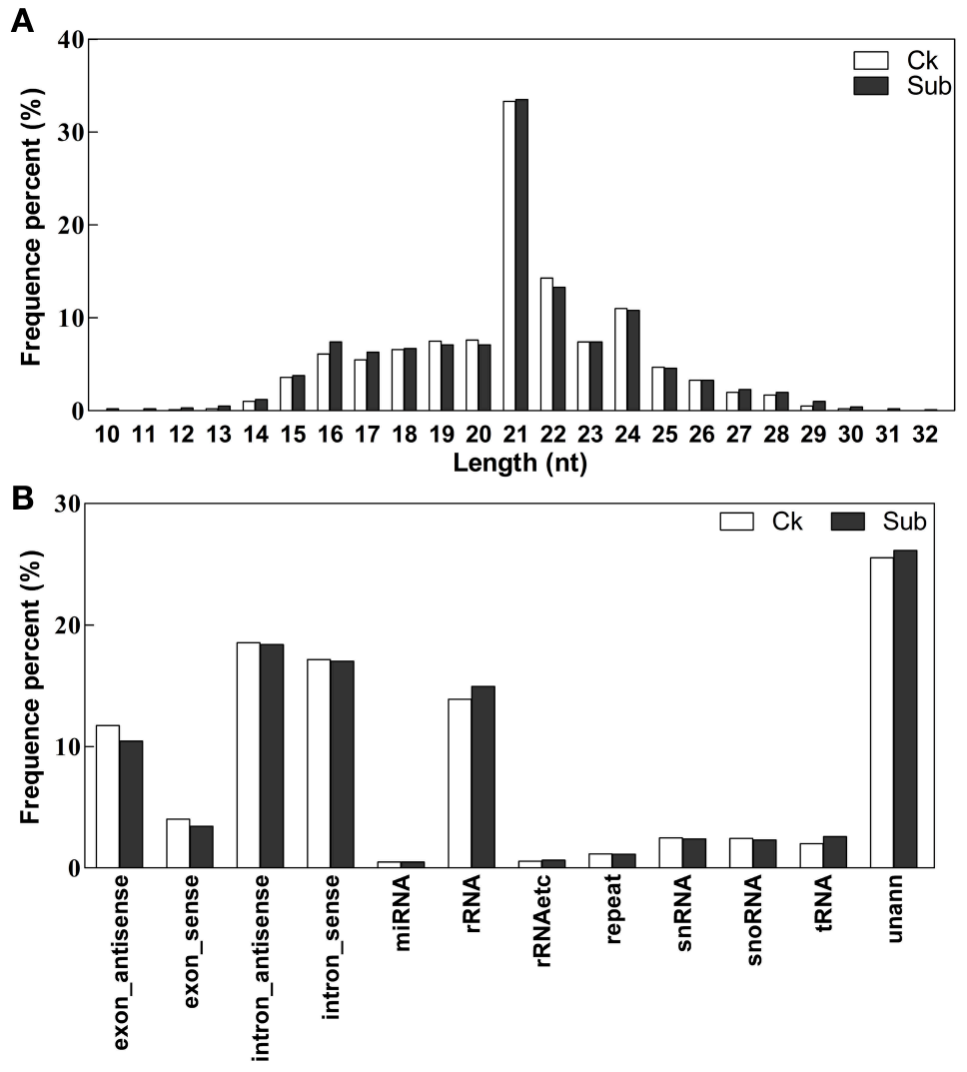
**Length distribution (A)**, and composition **(B)** of the unique small RNA in Ck (control) and Sub (submergence) libraries.

To survey small RNAs in lotus and their role in plant response to complete submergence, two small RNA libraries, with (Sub) and without (Ck) submergence-treatment, were sequenced by Illumina sequencing technology. The raw reads were filtered using SOAPnuke to cut adaptor sequences, remove low quality reads and contamination sequences of adaptors and finally we obtained 12,448,082 and 12,922,303 clean reads from the Ck and Sub libraries respectively (Table S2). It is well accepted that 21–24 nucleotides is the common length of functional small RNAs (Bo et al., 2009). The results of size distribution of unique small RNAs in the two libraries (Figure 1A) showed that the majority of length of the obtained sRNA sequences was 21–24 nt long, which is consistent with the typical size range of Dicer-processed products. Instead of 24 nt sRNAs, which was the most abundant sRNA in previous work (Fahlgren et al., 2007; Rajagopalan et al., 2007), 21 nt sRNAs was dominant in both our libraries. When mapping the clean reads of both libraries to various publically known non-coding RNA databases, there was no obvious difference of the percentage of each type of non-coding RNAs (Figure 1B, Table S2). In our mapping results, 2,314,221 sequences (9378 unique sequences) of Ck library and 2,722,313 sequences (9532 unique sequences) of Sub library were similar to known miRNAs (miRBase 21) identified in other plant species. This account for about 0.5% of unique clean reads and 20% of total clean reads in both small RNA libraries. In addition, about 25% of unique sequences could not map to any database. These sequences might possibly contain novel miRNA candidates and other classes of small RNAs.

### Known miRNAs and evolutionary conservation

A total of 758 known miRNAs belonging to 147 families were identified in the two libraries (Table S3) by aligning with miRBase 21 as described previously (Cheah et al., 2015). Overall, the miR156 family has the most abundant members in lotus while most miRNA families have only one member (39%). Based on the expression level of known miRNA families, they were assigned to three categories including low (TPM <100), moderate (TPM = 100–10,000) and high (TPM > 10,000) (Table S3; Cheah et al., 2015). For instance, NNU_aly-miR168a-5p, an important miRNA that is associated with plant development regulation (Xian et al., 2014), was classified into the high group in both libraries. By contrast, five members in lotus miR395 family belonged to the low expression category (TPM <100), which was consistent with the findings of Mallory and Vaucheret (2006) that miR395 was not detectable in plant under normal growth condition, unless it was stressed by low-sulfate or low-phosphate.

**Figure 2:**
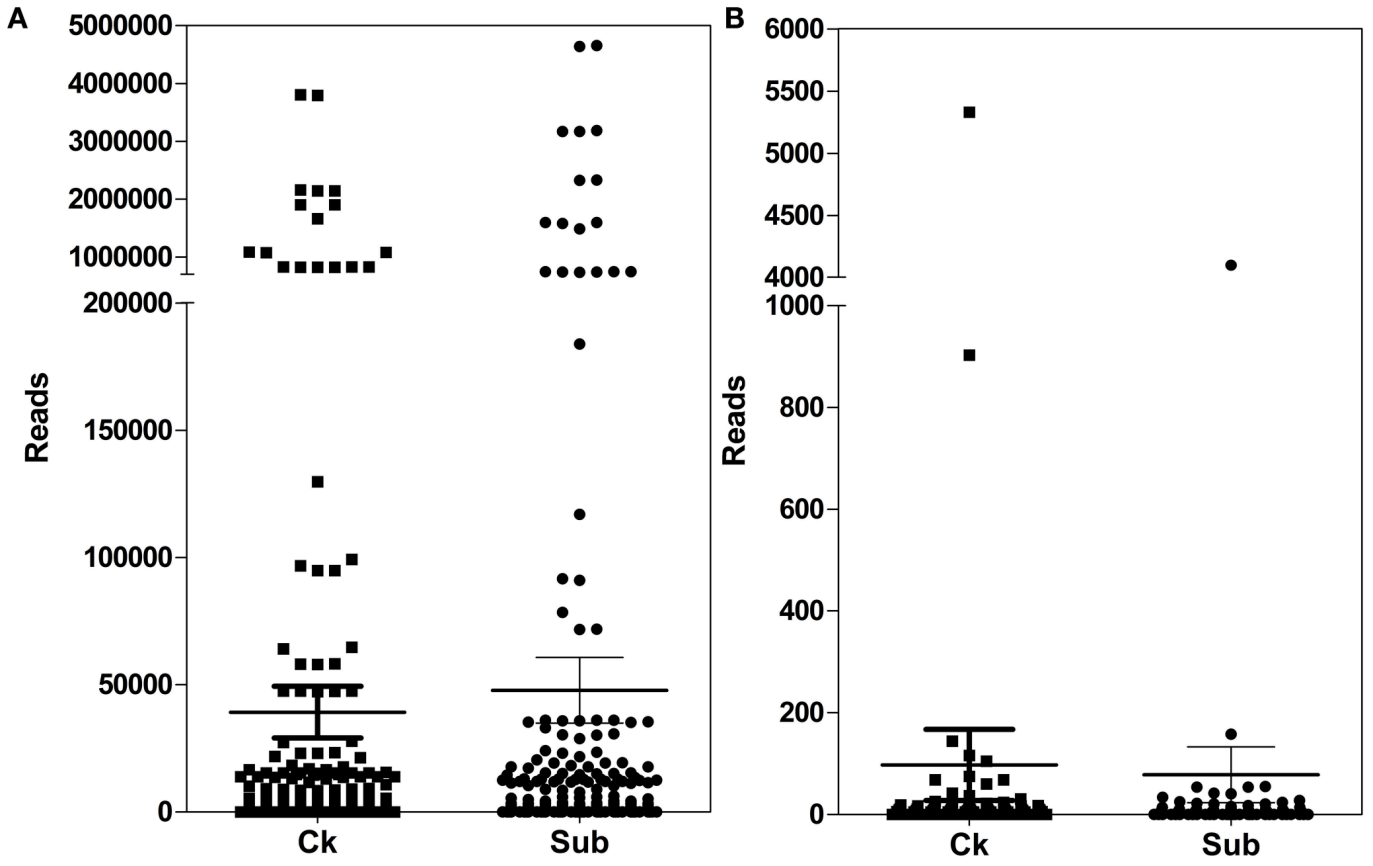
**Abundance of known (A)** and novel **(B)** miRNAs in lotus in Ck (control) and Sub (submergence) treatment.

The number of reads of detected miRNAs was shown in Figure 2. These miRNAs showed a large divergence in expression level (Figure 2). The read counts of known miRNAs varied from 0 to 4,659,812. miR166 family was largely enriched in both sequencing datasets. As reported previously, miR166 family was also abundant in other plant species, i.e., Arabidopsis, black pepper (Zhu et al., 2011; Asha et al., 2013).

It was also observed that different miRNA members in the same family showed large differences in their abundance. For example, although the miR162 family was demonstrated to be highly expressed in this work, only NNU_bdi-miR162, one of the six members in the miR162 family, was found to be highly expressed (TPM > 10,000) while expression of NNU_zma-miR162-5p was very low in both libraries (TPM <100). miR529 was another abundantly expressed miRNA family in both libraries, which has three members. Within the miR529 family, NNU_aqc-miR529, and NNU_far-miR529 (TPM > 10,000) were highly expressed, while NNU_osa-miR529b (TPM <10) was weakly expressed. These results suggested that our transcriptome analysis is sensitive enough to differentiate the expression of different members within a miRNA family.

The known miRNAs were then used to estimate their evolutionary roles by comparing them against known miRNAs in 63 other plant species (Table S4, Figure S3). Out of 147 lotus miRNA families, 103 were not highly conserved, as only one ortholog was noted in the other plant species (Yang et al., 2013). Five known lotus miRNAs were highly evolutionarily conserved with orthologs in over 20 plants from different divisions, indicating that these miRNAs might serve important functions in the plant kingdom. It is interesting to note that some deeply conserved miRNAs i.e., miR166, miR319, miR390, and miR171, in higher plants were also conserved in lower plants i.e., *Physcomitrella patens* (Arazi et al., 2005).

### Identification of novel miRNAs

Identification of stable hairpin structure of pre-miRNAs is key to predict novel miRNAs (Ambros et al., 2003). Thus, we analyzed hairpin structures of the putative miRNA candidates using Mireap software (Li et al., 2012) and identified 152 putative miRNA candidates whose precursors could form stable secondary structures as described (Meyers et al., 2008). Among them, miRNA^*^ were detected for 77 miRNA candidates. It is well accepted that the presence of corresponding miRNA^*^ can provide evidence that they are cleaved from pre-miRNA by DCL1 enzymes (Meyers et al., 2008). Thus, we considered those with identified miRNA^*^ as novel miRNA candidates (Table S5). As with many other novel miRNAs identified in a variety of species (Zhang et al., 2008; Yang et al., 2013; Jin et al., 2015; Xu et al., 2015), the expression of most novel miRNAs identified in this work was also found to be very low (TPM <100). About 94.81% of the novel miRNA candidates were classified having a low expression level (TPM <100) and a few novel miRNAs had a TPM over 100 (3.25%) or 1000 (1.95%).

### Target analysis of novel and known miRNAs by degradome

Out of 758 known miRNAs, 715 miRNAs were predicted to target 3332 potential target genes (Table S6). We also identified 232 targets genes for 64 novel miRNA candidates. The result showed that most miRNAs have more than one predicted target, i.e., novel_mir_52 having 25 predicted targets (Table S6). No target gene was predicted for 43 known miRNAs and 13 novel miRNAs, which might be due to limitations of the available genome data or because there is no actual target. To obtain the comprehensive annotation of these genes, all putative target transcripts in both libraries were annotated using Gene Ontology (GO) terms (Table S7). All the target genes were separated into numerous functional categories. It was also seen that some target genes were poorly characterized, suggested that these genes might play previously undescribed role in lotus.

The verification of miRNA targets provides supporting evidence for the existence of predicted novel and known miRNAs. It has been well documented that miRNAs cleave complementary target genes at the site correspond to its 10th nucleotide and produce many more sequence fragments with their 5′-end being complementary with the miRNA from the cleaved site (Addo-Quaye et al., 2008; German et al., 2008). A recently developed degradome sequencing technology made it possible to massively identify these cleavage products, which provides further experimental evidence for the existence of miRNA:target pairs (Barrerafigueroa et al., 2012). In this work, CleaveLand pipeline (Addo-Quaye et al., 2008, 2009) was used to identify cleaved targets for miRNAs in lotus using the available degradome dataset from NCBI (SRX1598165). All the degradome sequence tags were mapped to each lotus gene transcripts and targets were identified according to established criteria (Addo-Quaye et al., 2008, 2009). The abundance of the degradome sequence tags plotted on identified target genes is shown in Table S8. These target genes were classified into five categories (categories 0, 1, 2, 3, and 4) according to the abundance of mapped degradome sequence tags (Addo-Quaye et al., 2009). Fifty-six target genes have been divided into category 0 and 1, meaning targets are transcripts where the degradome tags corresponding to the expected miRNA-mediated cleavage sites were the most abundant tags matching the transcript. For known miRNAs, we were able to confirm 437 targets for 508 miRNA candidates (Table S8). About one-third of the known miRNAs without any validated target genes, expressed at a very low level, i.e., NNU_aly-miR3434-3p and NNU_aly-miR3436-5p which may, in part, explain the absence of cleaved targets. A large number of target genes were transcription factors, such as the AP2-like ethylene-responsive transcription factor, *GAMYB*, and *WRKY* (Tables S3, S8). Some target genes of known miRNAs were evolutionary conserved in different plants, including lotus. In Arabidopsis and tomato, miR160 was reported to target auxin-response factor (ARF) genes (Rhoades et al., 2002; Itaya et al., 2008) which were also the potential target genes of NNU_ahy-miR160-5p, suggesting a conserved role of miR160 in lotus.

Out of the 77 novel miRNAs with star strands, we confirmed 49 targets for 34 novel miRNAs (Tables S5, S8). We also showed the sequence tags from the degradome on target transcript sequences. The results showed that the overall abundance of mapped tags is lower than the known miRNAs and some targets of these novel miRNAs were difficult to detect. Low expression levels might lead to small amount of confirmed target genes. It should be noted that the target genes that could not be validated by degradome data might silence genes by repressing translation.

### Submergence-responsive small RNAs in lotus

**Figure 3:**
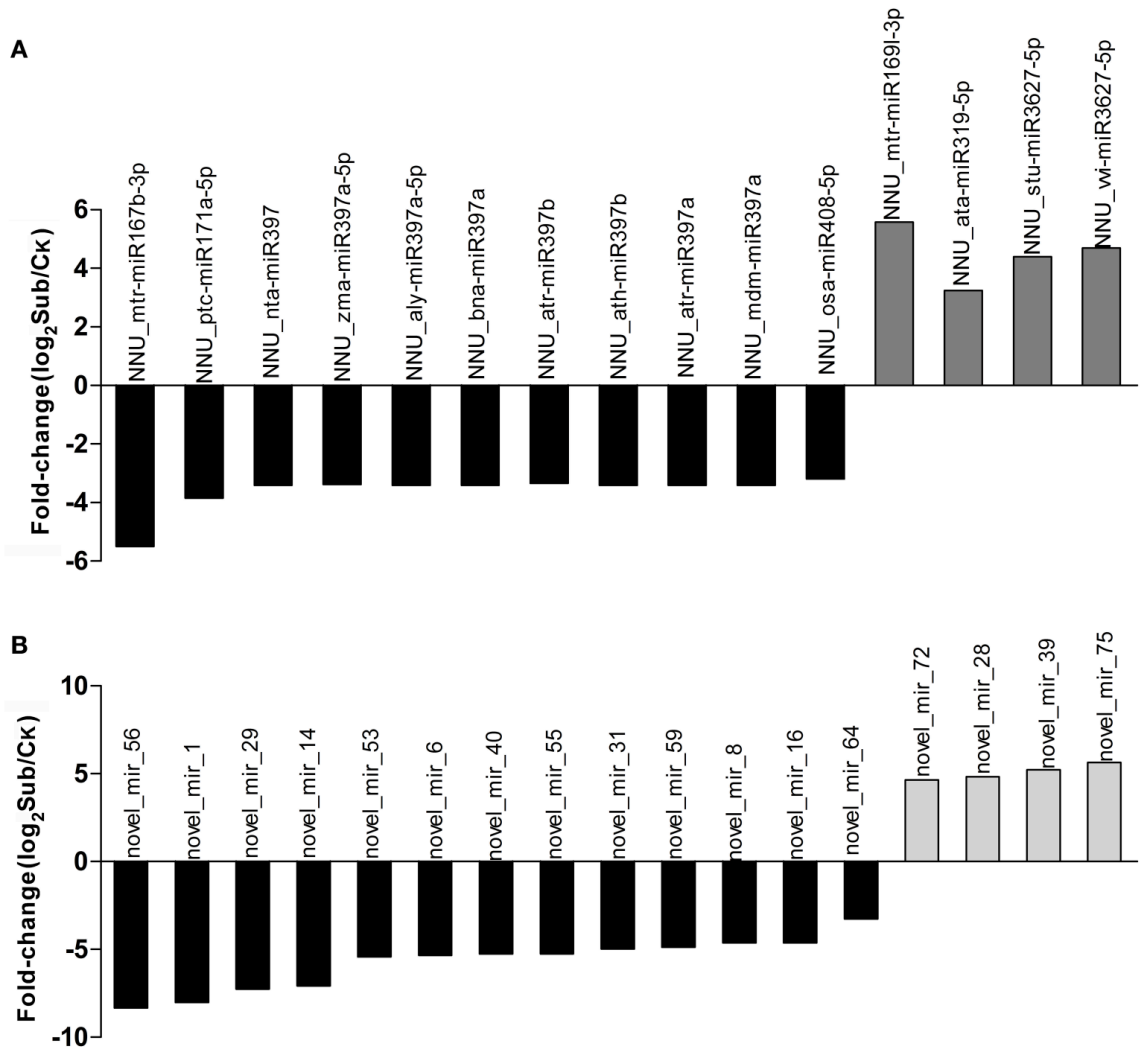
**Differential expression of significantly changed known (A)** and novel **(B)** miRNAs (>3-fold) comparing Sub (submergence treatment) vs. Ck (control) libraries.

Distinctive expression patterns of miRNAs between Ck and Sub libraries offered an opportunity to identify some important miRNAs that were functionally responsive to submergence stress. Results showed that 85.10% of total sRNAs were shared between the Ck and Sub libraries, which however only account for 10.81% of the total unique sRNAs (Figure S4). By contrast, most sRNAs which expressed in only one of the libraries showed low expression levels. Ck-specific unique sRNA (45.29%) was slightly higher than Sub-specific unique sRNAs (43.90%), which suggested that more genes were induced under submergence. We then made a comparative expression analysis of miRNAs between Ck and Sub libraries (Tables S3, S5). Results showed that many miRNAs were differentially expressed in lotus when upon submergence. In most cases, there was a decrease in miRNA frequency, with only 33.78% of significantly changed miRNAs experiencing an increase in expression. In known miRNAs, 46 miRNAs were significantly (fold change ≥ 1 and *Q* ≤ 0.001) up-regulated and 82 were down-regulated after submergence treatment, which indicated that more genes were up-regulated during submergence treatment. Twelve and 22 known miRNAs were only expressed in the control and submergence treatment libraries, respectively. Fifteen known miRNAs were clearly changed with log_2_ fold change>3 (Figure 3A). Apart from a small number of miRNAs, multiple members of the same miRNA family showed similar expression patterns (e.g., miR159, miR168, and miR393 family). Some previously reported stress-related miRNAs, i.e., miR393, miR397b, and miR402, that are known to be important for plant response to abscisic acid (ABA), cold, dehydration and salt stress, exhibited no significant changes in lotus upon submergence (Sunkar and Zhu, 2004). The inconsistency of these miRNAs' expression patterns suggested that a set of specific miRNAs might involve in lotus submergence response. In accordance with this suggestion, we found 20 novel miRNAs (25.97%) that were differentially expressed in control and submergence treatment seedlings. Sixteen of these novel miRNAs were up-regulated by submergence (Table S5, Figure 3B). Among all the identified miRNAs, novel_mir_75 showed the strongest induction and novel_mir_56 the strongest repression in response to submergence.

**Figure 4:**
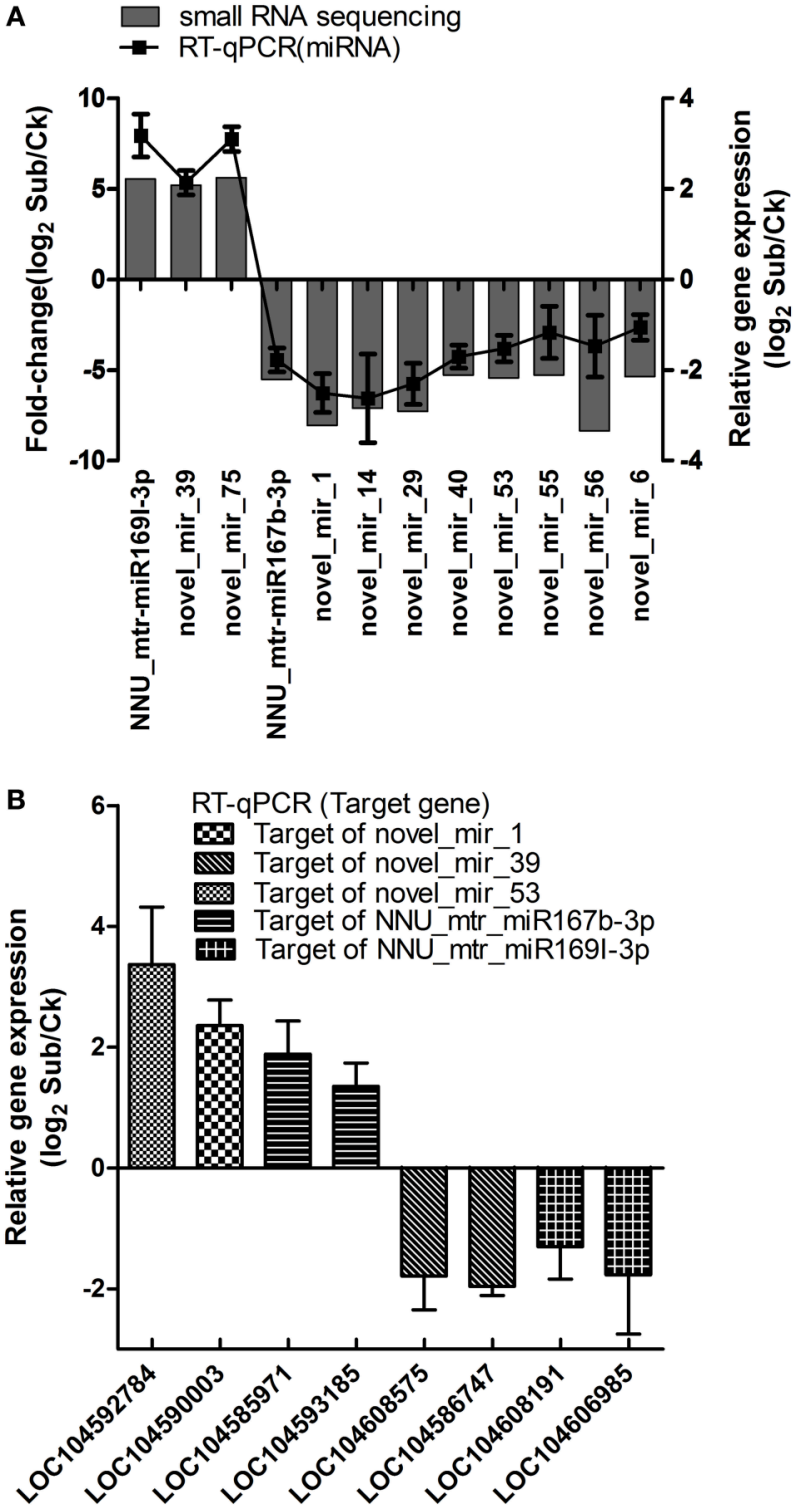
**Validation of differentially expressed miRNAs (A)** and corresponding target genes **(B)** using RT-qPCR comparing Sub (submergence treatment) vs. Ck (control) in lotus. Data are mean ± SE of four independent experiments.

To confirm the results obtained from small RNA deep sequencing, the expression patterns of 12 submergence responsive miRNAs (fold changes>5) including two known miRNAs and 10 novel miRNAs were analyzed by RT-qPCR (Figure 4A). As expected, the expression pattern of 12 selected miRNAs obtained from RT-qPCR was similar in magnitude to those obtained by deep sequencing. We also examined the expression patterns of eight selected target genes of miRNAs which were validated by transcriptome. As shown in Figure 4B, miRNA-mediated regulation of target gene expression level appears to be occurring. These results suggesting that the data from sRNA and transcriptome sequencing are reliable to be used to investigate submergence-induced miRNAs and target genes in lotus.

### The potential target genes of submergence-responsive miRNAs

**Figure 5:**
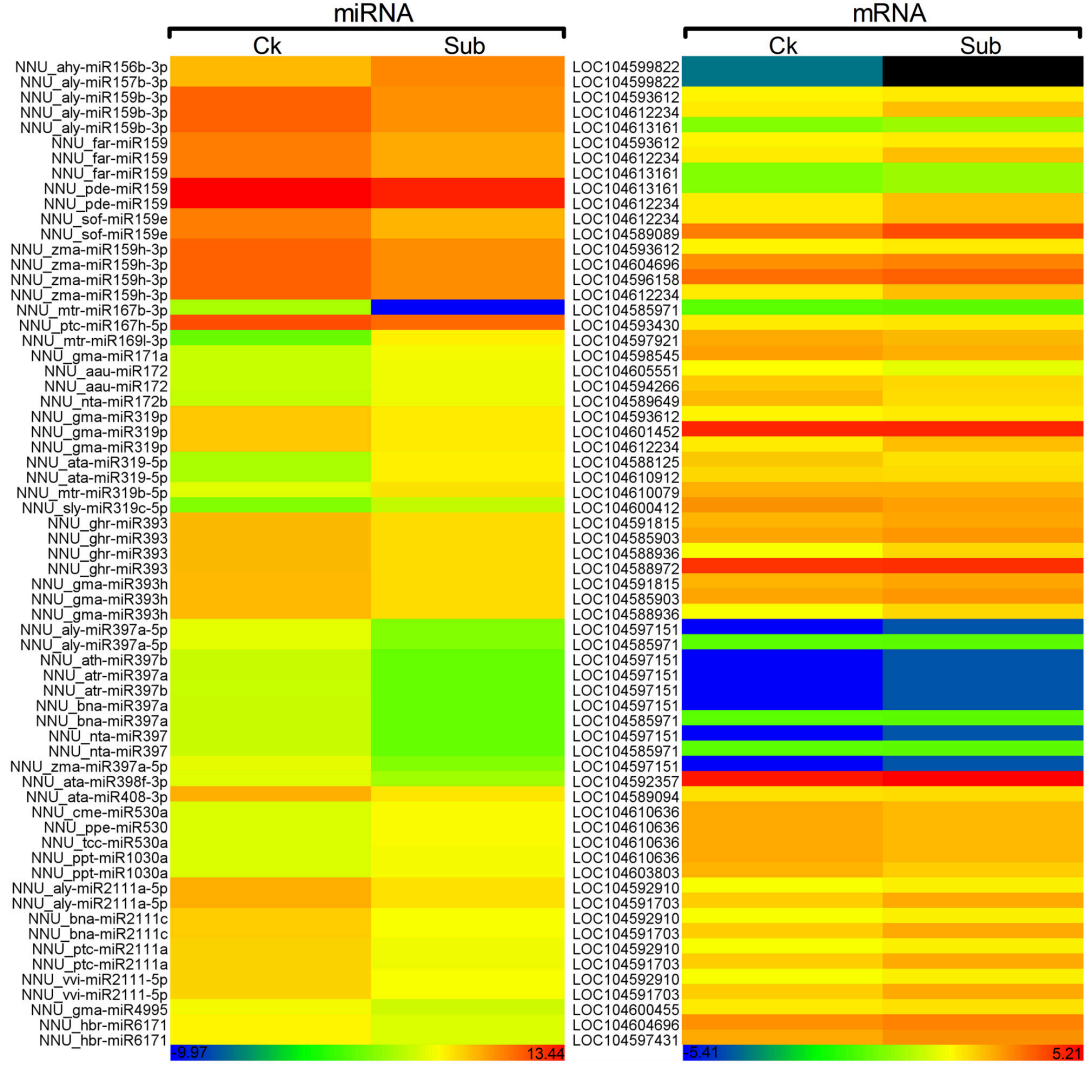
**Heat map of expression of submergence-responsive miRNAs and corresponding target genes which were validated by degradome and transcriptome**. Color scale represents normalized log_2_ transformed counts. Blue indicates low expression and red indicates high expression. Black indicates the genes that were not been detected.

**Figure 6:**
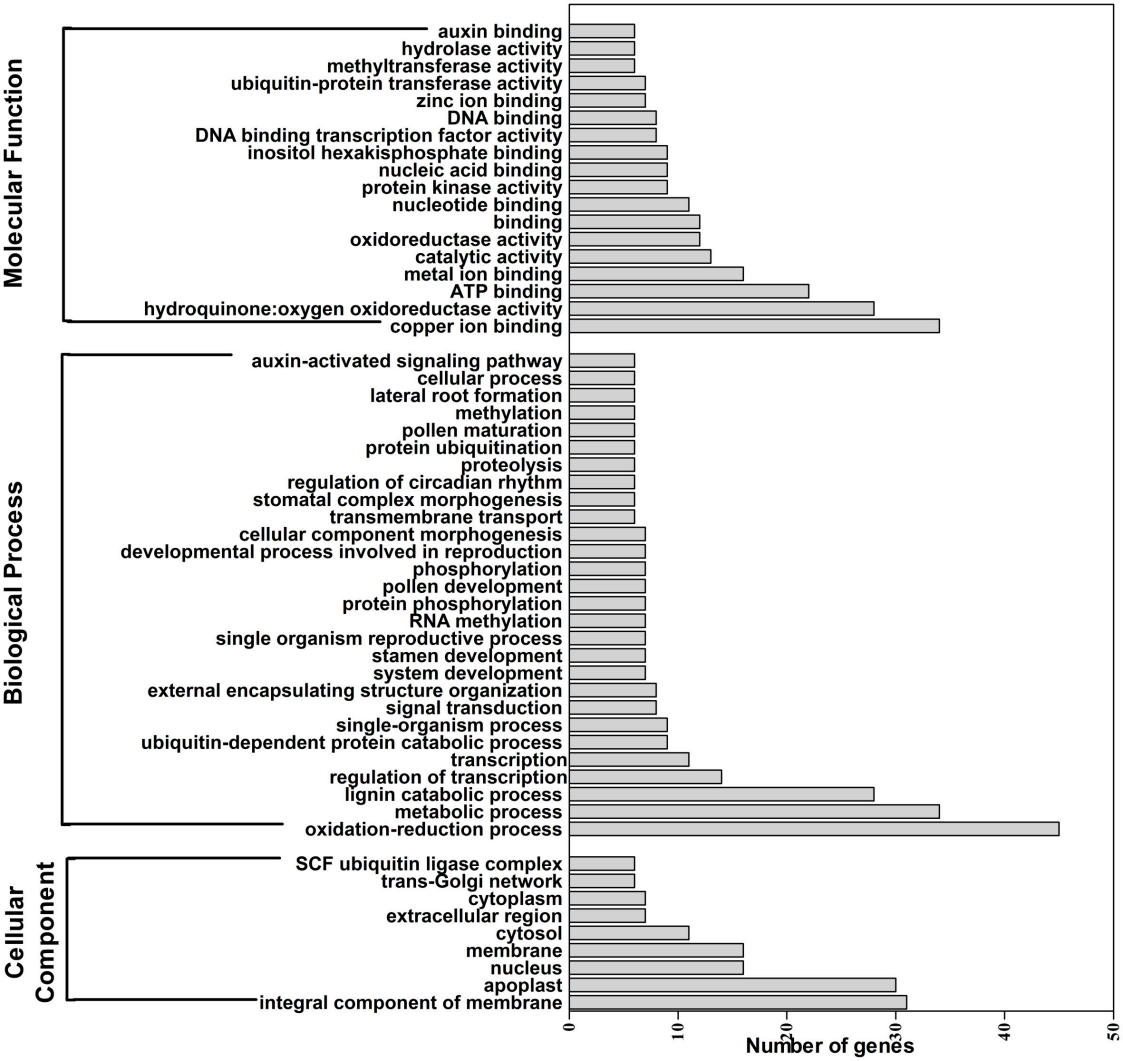
**GO classification of target genes for submergence responsive miRNAs identified in lotus**. The number of genes for each Gene Ontology (GO) term from each gene category.

**Figure 7:**
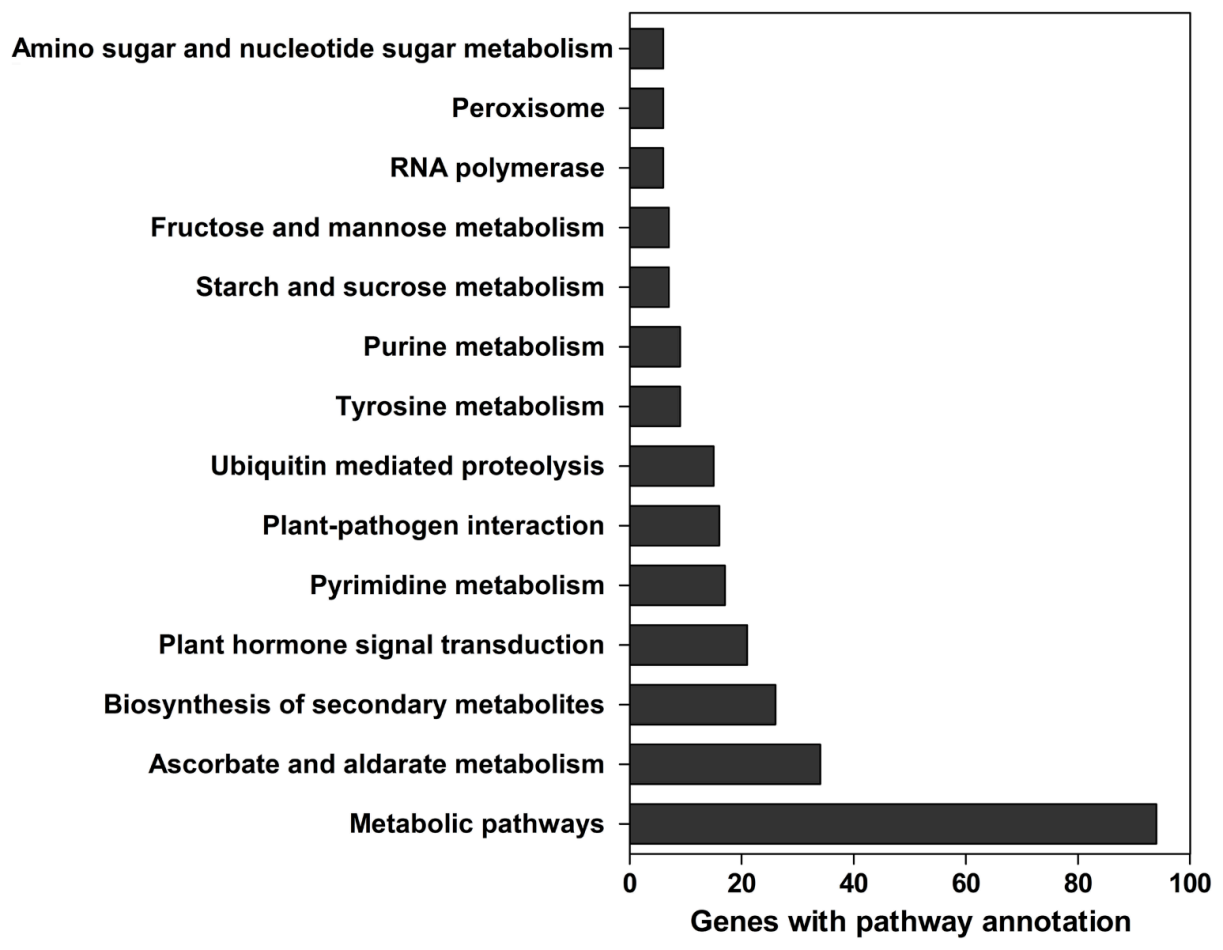
**The most enriched KEGG pathways of target genes for differentially expressed miRNAs**.

A total of 629 potential target genes were identified for submergence responsive miRNAs. Some genes were targeted by more than one miRNA. Taking advantage of the previously available transcriptome data of lotus under the same submergence treatment condition, we analyzed the expression level of those target genes to screen the genes that showed negative regulation pattern of corresponding miRNAs (Table S9). Of the 629 target genes, 290 were confirmed to be negatively regulated by 124 miRNAs. Degradome data provided evidence for the regulatory effect of submergence responsive miRNAs on 65 targets genes (Figure 5, Table S9). As shown in Table S9, Figure 5, the regulatory relationships of a large number of miRNA:target pairs could not be confirmed by transcriptome data. However, it may not be that these target genes were falsely predicted, but rather that beyond miRNAs they may also be regulated by other factors such as transcription factors and epigenetic events. Thus, in the following analysis, all the 290 transcriptome confirmed target genes were used. The biological function of the gene targets from the 290 genes negatively regulated in response to submergence were performed by blast2GO analysis. As shown in Figure 6, 18 molecular functions, nine cellular components categories and 28 biological processes are frequently involved in plant submergence responses. Many studies have indicated that under complete submergence, lotus, and some other semiaquatic plants, i.e., deep-rice could enable submerged petioles or shoots to quickly emerge from water, thus restoring gas exchange (Voesenek et al., 2004). To achieve this, lotus must promote carbohydrate metabolism and activate related proteins for petiole elongation. Consistent with this, we can see from Figure 6, the most abundant GO categories of biological processes were metabolic processes and oxidation-reduction. Lignin catabolic process was specifically activated upon submergence. A previous paper in deep-rice also showed that submergence-induced elongation of rice shoot decreased the structural carbohydrate level and lignin content were negatively associated with shoot elongation (Panda et al., 2013). Moreover, complete submergence also poses another severe problem in lotus; impaired mitochondrial respiration can induce overproduction of reactive oxygen species (ROS) resulting in oxidative stress and cell damage (Dutilleul et al., 2003). Thus, to reestablish the homeostasis of ROS, plant cells activate more target genes related to oxidation-reduction processes, which is also consistent with our observation from GO annotation. Moreover, these target genes were also annotated in multiple KEGG pathways (Figure 7). Among them, the metabolic pathways and ascorbate and aldarate metabolism might play important roles in regulating lotus petiole elongation and maintaining ROS homeostasis.

### Submergence responsive miRNAs regulate metabolic and morphological adaptations

Changes in miRNAs expressions enable plants to respond to oxygen limitation with a significant reprogramming of gene expression. Submergence resulted in the significant decrease in expression of 82 known miRNAs and 16 novel miRNAs under submergence, indicating that miRNAs were mainly involved in activating gene expression under submergence. Based on the gene targets, which are negative regulated by miRNAs as noted above, the miRNA-mediated regulatory networks in lotus response to submergence were constructed by using Cytoscape (Figure S5). NNU_aly-miR397a-5p, NNU_aly-miR159b-3p, NNU_mtr-miR167b-3p, NNU_gma-miR319p, and NNU_hbr-miR6171 regulated 52 different genes and constructed the most complicated regulatory network in this work (Figure S6). It is interesting to note that all the miRNAs in this subnetwork were down-regulated in response to submergence. Some target genes, especially those that were regulated by multiple miRNAs, could be confirmed by degradome data. According to GO term of these target genes, it was seen that this subnetwork mainly participated in three biological processes including oxidation-reduction process, lignin catabolic process, and metabolic process (Figure S6). KEGG pathway analysis showed that they were involved in metabolic pathways, ascorbate, aldarate metabolism, steroid biosynthesis, and cell cycle (Figure S6). These results point to the possibility that the miRNAs and corresponding target genes in this subnetwork might play an important role in regulating metabolic and morphological adaptions of lotus. In some cases, one target is shared by two or more miRNAs. For example, LOC104601452 is regulated by NNU_gma-miR319p, NNU_aly-miR319c-3p, and NNU_aqc-miR159.

**Figure 8:**
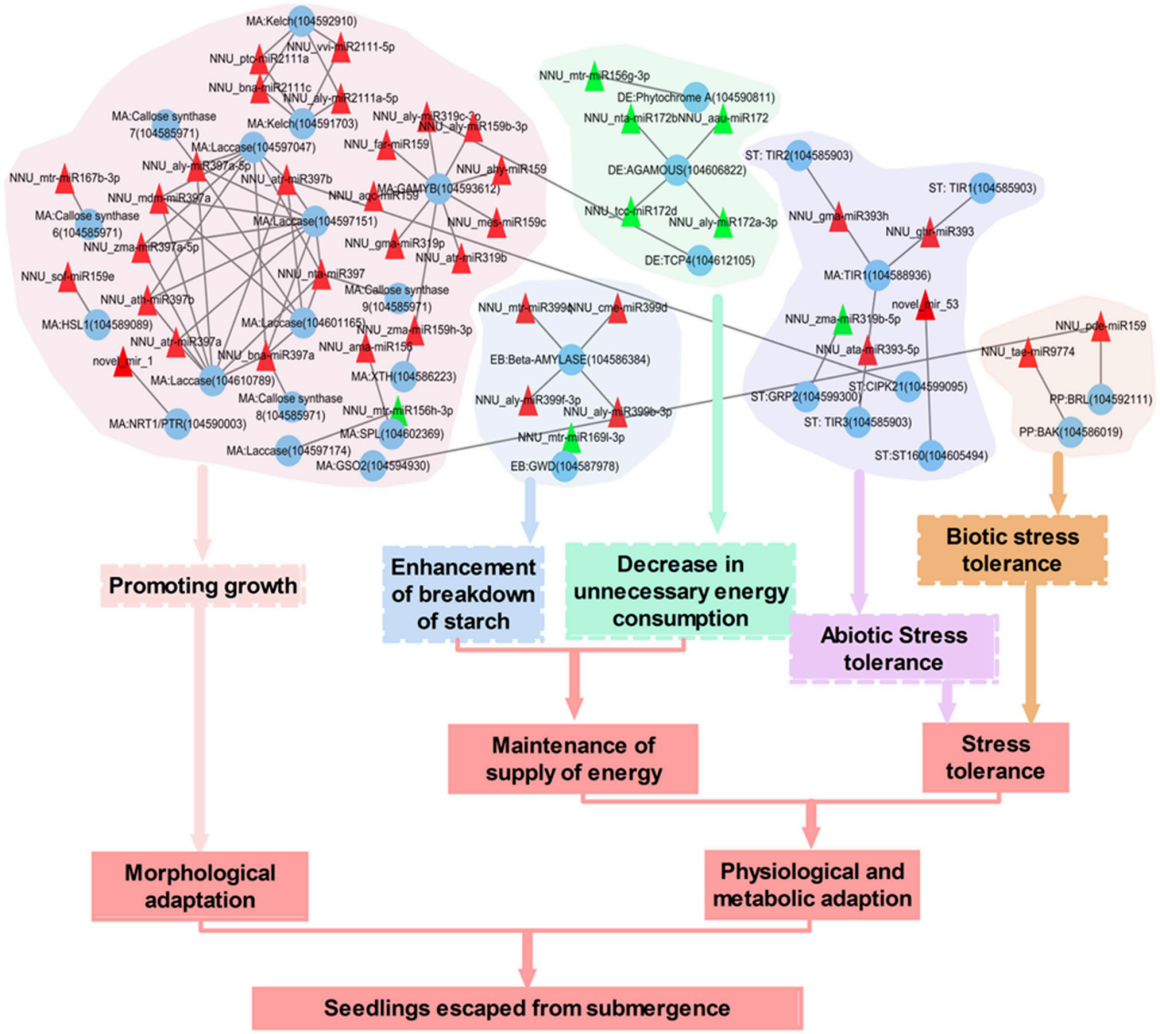
**The potential regulating network of submergence-responsive miRNAs in lotus**. Red triangle, down-regulated miRNAs; Green triangle, up regulated miRNAs. Blue circle, mRNAs. MA, Morphological adaptation; ST, Stress tolerance; EB, Enhancement of breakdown of starch; DE, Decrease in biosynthesis of starch; PP, Plant-pathogen interaction.

Based on regulatory networks, functional annotations and literature mining, we found some submergence-related evidence for the miRNAs and target genes (Figure 8). Elongating submerged petiole to emerge from water and thus restoring gas exchange was an important mechanism of lotus to escape from submergence stress. Several phytohormones including ethylene, auxin, GA, and ABA were reported to be involved in this process (Cox et al., 2004). Here, we found some miRNAs which directly altered the level of transcripts encoding some important component in those phytohormones regulated networks were significantly changed in response to submergence. For instance, several members from miR159 and miR319 family constructed a subnetwork to control the expression of *GAMYB* genes which mediated GA signaling in petiole elongation (Gocal et al., 2002). All the miRNAs in this network were down-regulated in response to submergence, which resulted in the up-regulation of *GAMYB* gene and might promote petiole elongation. Four miRNA (NNU_aly-miR159b-3p, NNU_far-miR159, NNU_gma-miR319p, and NNU_zma-miR159h-3p):target (GAMYB) pairs were confirmed by degradome data (Table S9). Auxin was also reported to play a role in hyponastic growth of submerged *Rumex palustris* petioles (Cox et al., 2004). F-box proteins TRANSPORT INHIBITOR RESPONSE 1/AUXIN SIGNALING F-BOX (TIR1/AFB) are auxin receptors that mediate degradation of AUXIN/INDOLE-3-ACETIC ACID (Aux/IAA) repressors to induce auxin-regulated responses (Terrile et al., 2012). In the present study, the decreased expression of three miRNAs including NNU_ghr-miR393, NNU_gma-miR393h and NNU_ata-miR393-5p which targeting *TIR1* resulted in an increase of *TIR1* transcripts. The miRNA:target pairs of NNU_ghr-miR393 and NNU_gma-miR393h were validated by degradome. We also identified the change of a novel miRNA, novel_mir_1, which can facilitate the effect of those phytohormones (Chiba et al., 2015). The down regulation of novel_mir_1 increases the expression of an important transporter gene, *NRT1/ PTR*, which transports the plant hormones auxin (indole-3-acetic acid), and GA, as well as secondary metabolites (glucosinolates) (Chiba et al., 2015). Besides those genes, we also identified several genes which are responsible for cell division and elongation were induced with the down-regulating of corresponding regulatory miRNAs. Those genes including *GSO2* (Racolta et al., 2014), *HSL1* (Umeyama et al., 2005; Simpson-Lavy et al., 2009), *F-box/kelch-repeat protein* (Zhang et al., 2015), *laccase* (Ranocha et al., 2002), *SQUAMOSA PROMOTER BINDING PROTEIN-LIKE* (*SPL*) (Usami et al., 2009), and *xyloglucan endotransglucosylase/hydrolase* (*XTH*) (Saladié et al., 2006).

During complete submergence, energy supply becomes insufficient because aerobic respiration and photosynthesis are largely inhibited. Thus, maintenance of energy supply under submergence is an important factor for lotus to adapt to the stress. The down-regulation of several members in miR399 family resulted in accumulations of *Beta-AMYLASE*, which is required for starch breakdown (Fulton et al., 2008). Down regulation of NNU_csi-miR1515 enhanced carbohydrate metabolic process to cause more carbohydrate breakdown, which supplies the substrate for glycolysis to produce ATP. Meanwhile, the up-regulation of NNU_aly-miR319c-3p, NNU_mtr-miR156g-3p, NNU_aau-miR172, NNU_aly-miR172a-3p, NNU_nta-miR172b, and NNU_tcc-miR172d could decrease unnecessary energy consumption, i.e., flowering and light response, when under submergence. For example, *AGAMOUS* gene, which was down-regulated by four members of miR172 family, was involved in regulation of plant flowering time and floral organ identity (Aukerman and Sakai, 2004). *TCP4*, a target of NNU_aly-miR319c-3p is critical for petal growth and development (Nag et al., 2009). Phytochrome A signal was also down-regulated by the up-regulation of NNU_mtr-miR156g-3p (Yanovsky et al., 1997). When under submergence, these physiological processes waste limited plant energy. Thus, these miRNAs regulated enhancement of breakdown of starch and decreases in unnecessary energy consumption will help lotus to maintain its energy supply.

Besides morphological and metabolic adaptation, we also identified the changes of several miRNAs, which were associated with the enhancement of plant stress tolerance. miR393 has been identified as closely related to biotic and abiotic stresses and was found to target *TIR1* genes in this work. *TIR1* has been shown to have a role in enhancing plant stress tolerance (Chen et al., 2015). The down-regulation of NNU_ghr-miR393, NNU_gma-miR393h, and NNU_ata-miR393-5p resulted in increased expression level of *TIR1* genes. We also identified the accumulation of two other genes including *CIPK21* (Pandey et al., 2015) and *SR160* (Scheer et al., 2003) which could enhance lotus stress tolerance due to a decrease of NNU_atr-miR397b and novel_mir_53 respectively.

## Conclusion

In conclusion, the present study systematically analyzed the small RNA expression profiles of lotus upon complete submergence by using deep-sequencing, computational and molecular methods. A total of 147 known miRNA families were identified in lotus seedlings under normal or submergence condition. Using the unannotated small RNAs, 77 novel lotus-specific miRNAs with identified miRNA^*^ were predicted. Submergence treatment of lotus lead to 128 known and 20 potential novel miRNAs that displayed differential expression patterns. Moreover, 629 target transcripts were identified for submergence-response miRNAs. The miRNA:target pairs were confirmed by transcriptome and degradome data. Further characterization of submergence-related miRNAs and target genes provided deeper insight on lotus's response to submergence and an integrative model of miRNA-mediated regulatory network was presented (Figure 8). These results may contribute to a better understanding of lotus miRNAs in response to submergence stress.

## Author contributions

QJ and YX designed the experiments; QJ, BW, and XZ performed the experiments; QJ and BW did data analysis; QJ, YX, XL, BW, and YW drafted the manuscript; QJ, YX, NM, HJ, XJL, and DY reviewed and edited the manuscript. All authors read and approved the final manuscript.

## Funding

This work was supported by National Natural Science Foundation of China (31501795), the Fundamental Research Funds for the Central Universities (KJQN201659), the China Postdoctoral Science Foundation funded project (2014M560432, 2015T80563), Funding of agricultural science and technology innovation of Jiangsu Province, China (CX(15)1030, CX(16)1024), National Natural Science Foundation of China (31400600), and the Natural Science Foundation of Jiangsu Province in China, (BK20151229, BK20140695).

### Conflict of interest statement

The authors declare that the research was conducted in the absence of any commercial or financial relationships that could be construed as a potential conflict of interest.

## Supplementary material

Table S1 **List of primers used in this study**.

Table S2 **Statistics of small RNA sequences from Ck (control) and Sub (submergence treatment) libraries of lotus**.

Table S3 **Known miRNAs in lotus**. Identified lotus known miRNAs and their reads, sequences in Ck (control) and Sub (submergence treatment) libraries. Fold changes (Sub/Ck) and statistic results were also presented.

Table S4 **Evolutionary conservation of lotus known miRNA families**.

Table S5 **Novel miRNAs in lotus**. Identified lotus novel miRNAs and their reads, sequences, location in lotus genome, and structure of corresponding precursor in Ck (control) and Sub (submergence treatment) libraries. Fold changes (Sub/Ck) and statistic results were also presented.

Table S6 **Predicted targets of all miRNAs identified in lotus**.

Table S7 **Gene ontology classification of target genes**.

Table S8 **Target genes of lotus miRNAs verified b degradome data**.

Table S9 **Validation of target genes of submergence responsive miRNAs by transcriptome**.

Figure S1 **Flow chart of the methodology adopted to analyze small RNA in lotus**.

Figure S2 **Flow chart of the methodology adopted to analyze mRNA in lotus**.

Figure S3 **Known miRNA families in lotus and across-species**. The presence of miRNA families (y axis) in species (x axis) were show as red boxes.

Figure S4 **Common and specific sequences between Ck (control) and Sub (submergence treatment) library**.

Figure S5 **miRNA-regulated networks in lotus response to submergence stress**.

Figure S6 **miRNA-mediated gene regulatory subnetworks in response to submergence stress**. The target genes were annotated with GO term and KEGG pathway.
