# Supplementary material for: Identification of Submergence-Responsive MicroRNAs and Their Targets Reveals Complex MiRNA-Mediated Regulatory Networks in Lotus (Nelumbo nucifera Gaertn)
Source: Front Plant Sci. 2017 Jan 18;8:6. doi: 10.3389/fpls.2017.00006 (PMC5241310; doi:10.3389/fpls.2017.00006)
Supplement: Table S1 — List of primers used in this study. [file Table1.DOCX]

Table S1 List of primers used in this study.

| Primer name | Sequences (5’→3’) | Product  Length (bp) | Annealing Temperature (°C) |
| --- | --- | --- | --- |
| NNU_mtr-miR169I-3p | GGCAAGTTTTTCCTTGGCTATA |  | 56 |
| novel_mir_39 | TCCGTAAGATCATGGTGCCCGA |  | 56 |
| novel_mir_75 | GGGAGCTCCAGGCTCGCCCGAA |  | 56 |
| NNU_mtr-miR167b-3p | GATCATGTTGGAGCTTCACC |  | 56 |
| novel_mir_1 | GGCCTACAGCCCGAGGTGCTT |  | 56 |
| novel_mir_14 | GATATGCCAATCAGTGAGACCCTT |  | 56 |
| novel_mir_29 | GGTGTAACATTCCAGTTTTGCCCA |  | 56 |
| novel_mir_40 | GCCCTGTTGGTTCGAGGAGCCA |  | 56 |
| novel_mir_53 | GAATCCGAGCTCGGATGGATG |  | 56 |
| novel_mir_55 | CTTGTCAGAGGACAGGTGTCGGCA |  | 56 |
| novel_mir_56 | CTTAGTGTAAGTGTTGGGCCCATA |  | 56 |
| novel_mir_6 | GGTTCTGCTAACCCTGCGGTGA |  | 56 |
| Nn_U6-F | CCGATAAAATTGGAACGATACAG | 70 | 60.7 |
| Nn_U6-R | ATTTCTCGATTTGTGCGTGTCA |  |  |
| LOC104606985-F | GAGAAGATGGAGGTGCTAA | 102 | 58.8 |
| LOC104606985-R | ATCGGACAAGGACAAGTT |  |  |
| LOC104608191-F | CGCTATTCCAGTGATGAT | 111 | 58.4 |
| LOC104608191-R | GAGGCTTAGGTCTATTCC |  |  |
| LOC104593185-F | TCACACCTTCTAATAACCTCCAT | 125 | 59.6 |
| LOC104593185-R | ATCCTCACTGCCTCCTAATG |  |  |
| LOC104585971-F | GCGATTGTGCCGATTCTT | 188 | 80.1 |
| LOC104585971-R | TAACTTGTCTCCTCTTCTTCCTT |  |  |
| LOC104590003-F | AGGTGGAGGAGTTGAAGATAC | 157 | 80.2 |
| LOC104590003-R | AGAGATGCTGGAGGAATGG |  |  |
| LOC104592784-F | CGACATCTACAACCGAGGAT | 136 | 78 |
| LOC104592784-R | AACAAGAGAACCAGCATCAAG |  |  |
| LOC104586747-F | GAACGACAAGCACTCAGA | 180 | 60.1 |
| LOC104586747-R | TCCAGGTATAGTCCACAAGA |  |  |
| LOC104608575-F | ATGATGTTGGTGTCCGTGTT | 231 | 59.7 |
| LOC104608575-R | CTAAGGCACAAGCAGTAGCA |  |  |
| NnEF-1-F | TCGTCTGAATATGTGAATGTT | 190 | 59.2 |
| NnEF-1-R | CATCATCATCATCATCATCCT |  |  |
