# Supplementary material for: Identification of Submergence-Responsive MicroRNAs and Their Targets Reveals Complex MiRNA-Mediated Regulatory Networks in Lotus (Nelumbo nucifera Gaertn)
Source: Front Plant Sci. 2017 Jan 18;8:6. doi: 10.3389/fpls.2017.00006 (PMC5241310; doi:10.3389/fpls.2017.00006)
Supplement: Table S2 — Statistics of small RNA sequences from Ck (control) and Sub (submergence treatment) libraries of lotus. [file Table2.DOCX]

Table S2 Statistics of small RNA sequences from Ck and Sub libraries of lotus.

|  | Ck | |  |  | Sub | |
| --- | --- | --- | --- | --- | --- | --- |
| Type | Unique | Total |  | Type | Unique | Total |
| Raw reads |  | 14624355 |  | Raw reads |  | 15694869 |
| Clean reads | 1885372 | 12448082 |  | Clean reads | 1932986 | 12922303 |
| Match genome | 1418991 | 11129635 |  | Match genome | 1418901 | 11168777 |
| exon_antisense | 220938 | 478921 |  | exon_antisense | 202104 | 379626 |
| exon_sense | 75619 | 146628 |  | exon_sense | 66368 | 131278 |
| intron_antisense | 349316 | 1175120 |  | intron_antisense | 355358 | 1005338 |
| intron_sense | 323568 | 924880 |  | intron_sense | 329309 | 769949 |
| miRNA | 9378 | 2314221 |  | miRNA | 9532 | 2722313 |
| rRNA | 261760 | 4448174 |  | rRNA | 289240 | 4807400 |
| rRNAetc | 10527 | 473552 |  | rRNAetc | 12361 | 540445 |
| repeat | 22115 | 59180 |  | repeat | 22306 | 42456 |
| snRNA | 46872 | 130163 |  | snRNA | 46486 | 123935 |
| snoRNA | 46190 | 139157 |  | snoRNA | 44657 | 134798 |
| tRNA | 37703 | 486770 |  | tRNA | 49993 | 452633 |
| unann | 481386 | 1671316 |  | unann | 505272 | 1812132 |
