# Supplementary figures and images for: Identification of Submergence-Responsive MicroRNAs and Their Targets Reveals Complex MiRNA-Mediated Regulatory Networks in Lotus (Nelumbo nucifera Gaertn)
Source: Front Plant Sci. 2017 Jan 18;8:6. doi: 10.3389/fpls.2017.00006 (PMC5241310; doi:10.3389/fpls.2017.00006)

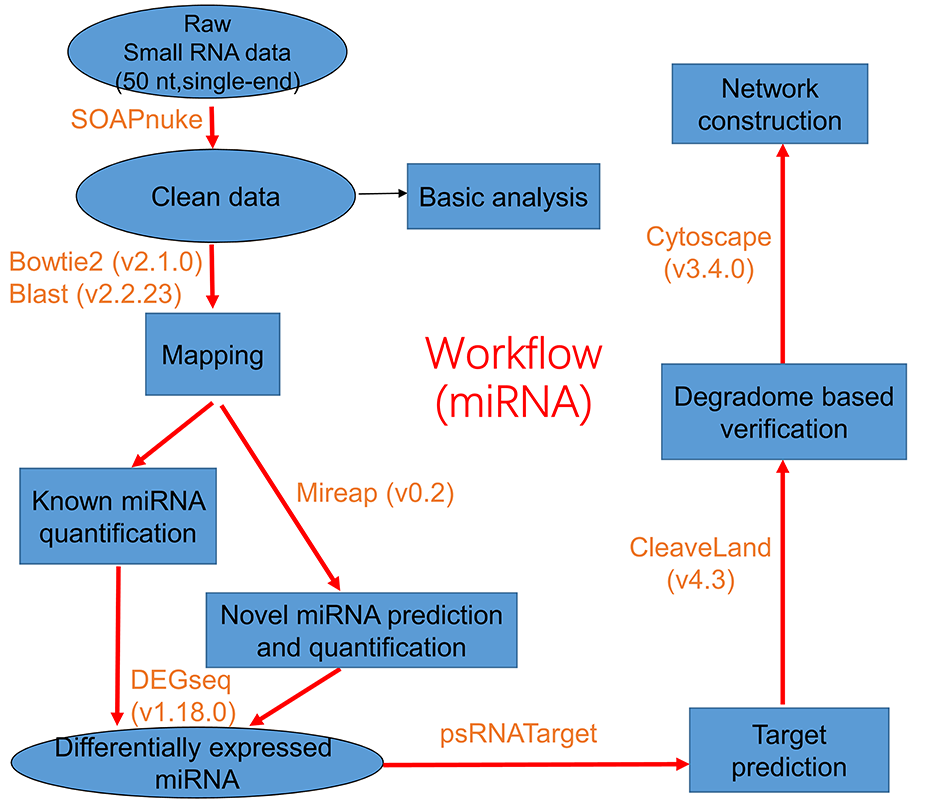

Supplement: Figure S1 — Flow chart of the methodology adopted to analyze small RNA in lotus. [file Image1.TIF]

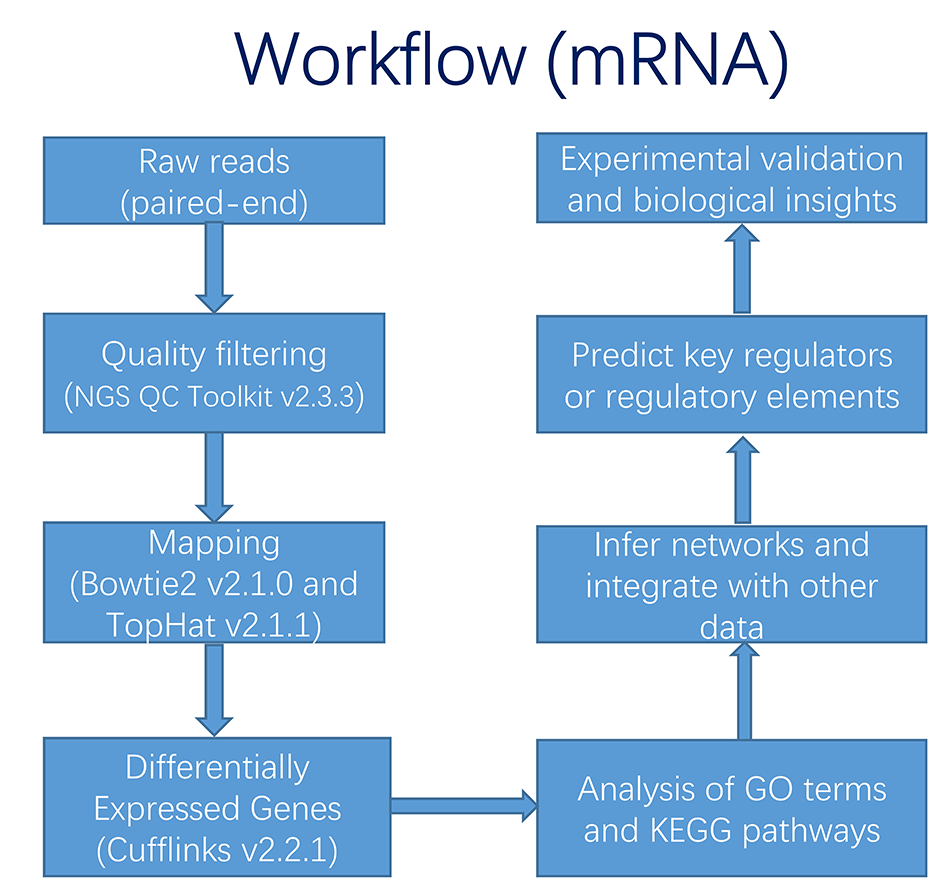

Supplement: Figure S2 — Flow chart of the methodology adopted to analyze mRNA in lotus. [file Image2.TIF]

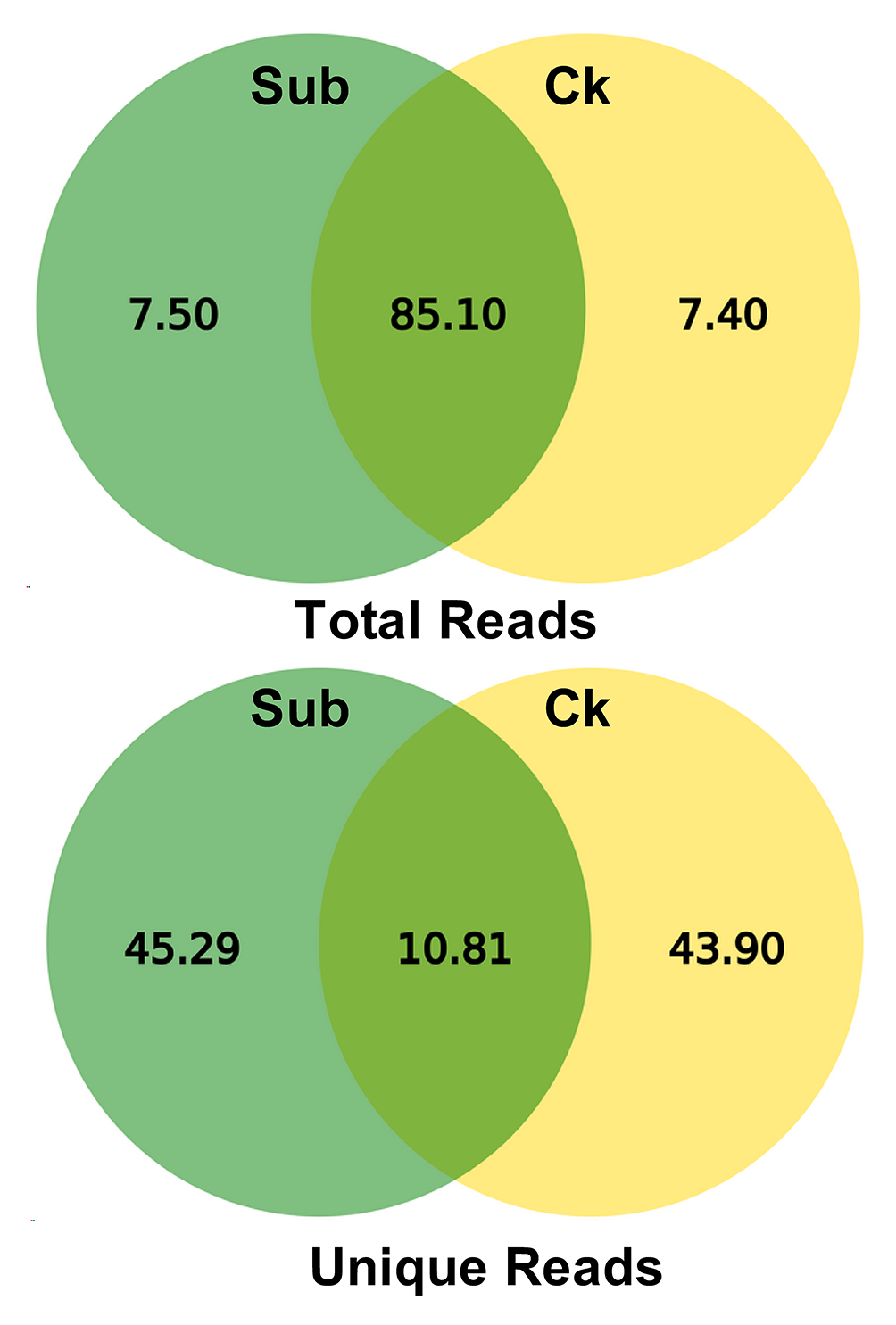

Supplement: Figure S4 — Common and specific sequences between Ck (control) and Sub (submergence treatment) library. [file Image4.TIF]

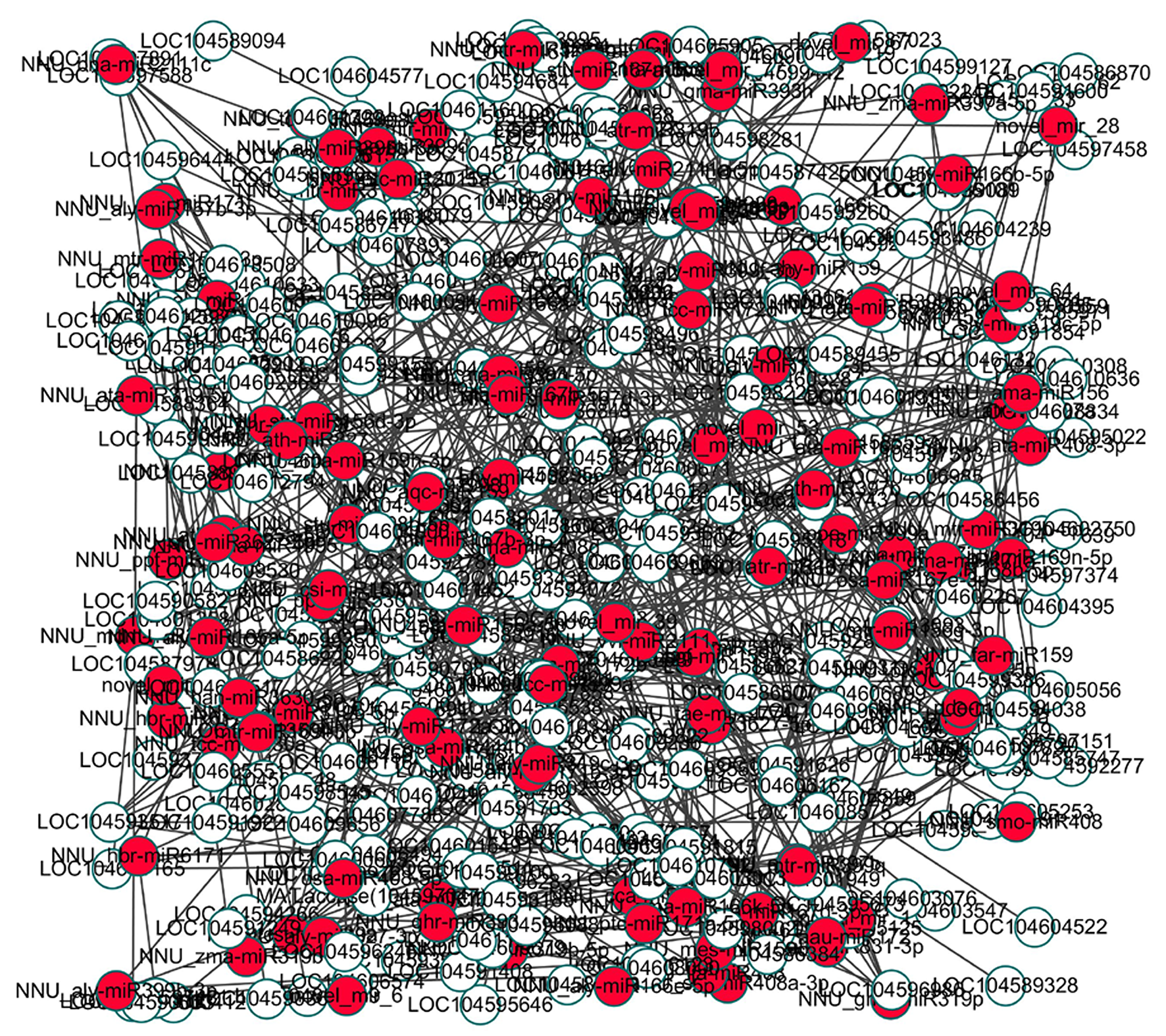

Supplement: Figure S5 — miRNA-regulated networks in lotus response to submergence stress. [file Image5.TIF]

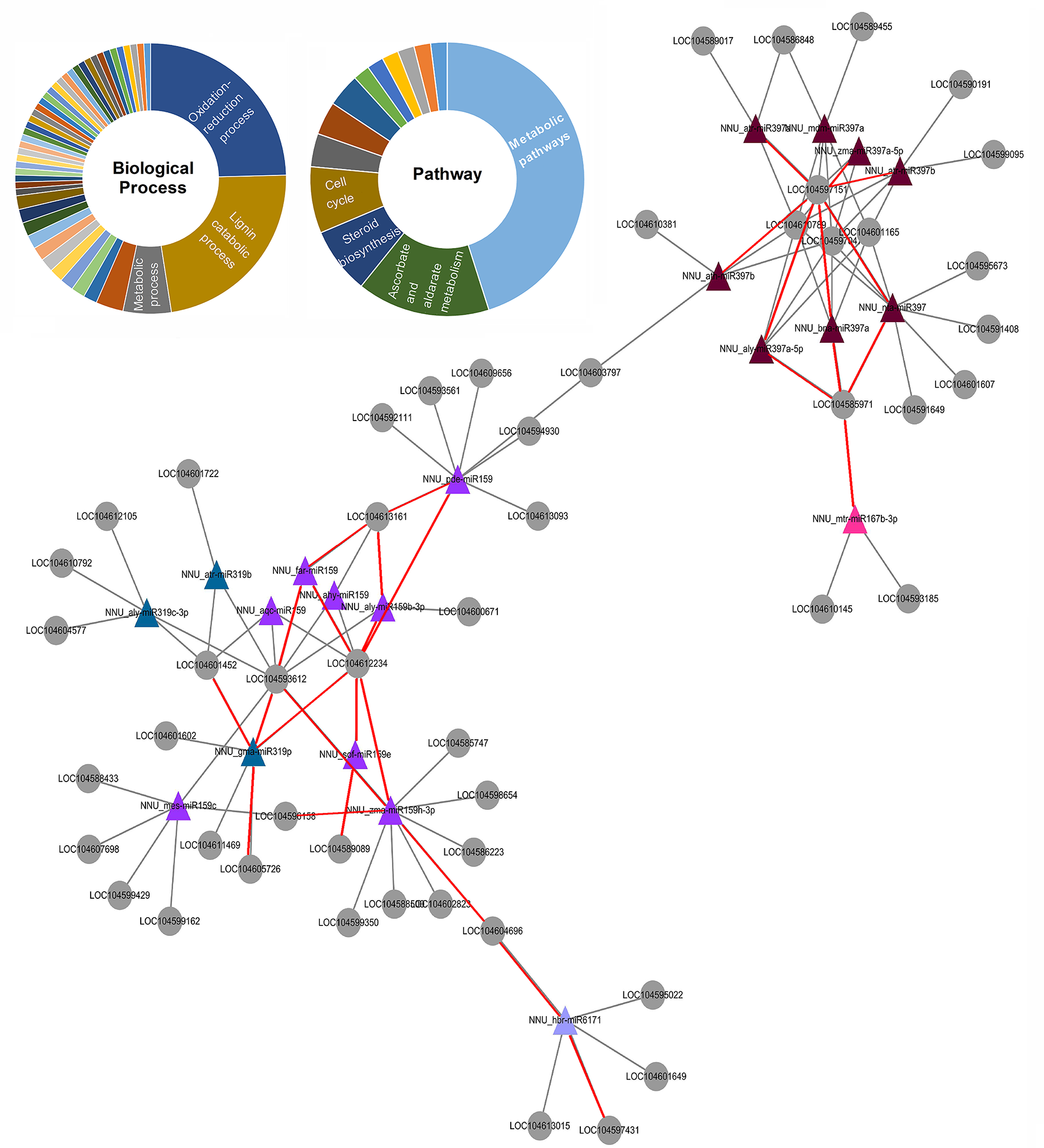

Supplement: Figure S6 — miRNA-mediated gene regulatory subnetworks in response to submergence stress. The target genes were annotated with GO term and KEGG pathway. [file Image6.TIF]
